# Myelin-associated glycoprotein modulates apoptosis of motoneurons during early postnatal development via NgR/p75^NTR^ receptor-mediated activation of RhoA signaling pathways

**DOI:** 10.1038/cddis.2015.228

**Published:** 2015-09-03

**Authors:** A Palandri, V R Salvador, J Wojnacki, A L Vivinetto, R L Schnaar, P H H Lopez

**Affiliations:** 1 Laboratorio de Neurobiología, Instituto de Investigación Médica Mercedes y Martin Ferreyra, INIMEC-CONICET-Universidad Nacional de Córdoba, Córdoba, Argentina; 2 Department of Pharmacology, Johns Hopkins University School of Medicine, Baltimore, MD, USA; 3 Department of Neuroscience, Johns Hopkins University School of Medicine, Baltimore, MD, USA; 4 Facultad de Psicología, Universidad Nacional de Córdoba, Córdoba, Argentina

## Abstract

Myelin-associated glycoprotein (MAG) is a minor constituent of nervous system myelin, selectively expressed on the periaxonal myelin wrap. By engaging multiple axonal receptors, including Nogo-receptors (NgRs), MAG exerts a nurturing and protective effect the axons it ensheaths. Pharmacological activation of NgRs has a modulatory role on p75^NTR^-dependent postnatal apoptosis of motoneurons (MNs). However, it is not clear whether this reflects a physiological role of NgRs in MN development. NgRs are part of a multimeric receptor complex, which includes p75^NTR^, Lingo-1 and gangliosides. Upon ligand binding, this multimeric complex activates RhoA/ROCK signaling in a p75^NTR^-dependent manner. The aim of this study was to analyze a possible modulatory role of MAG on MN apoptosis during postnatal development. A time course study showed that *Mag*-null mice suffer a loss of MNs during the first postnatal week. Also, these mice exhibited increased susceptibility in an animal model of p75^NTR^-dependent MN apoptosis induced by nerve-crush injury, which was prevented by treatment with a soluble form of MAG (MAG-Fc). The protective role of MAG was confirmed in *in vitro* models of p75^NTR^-dependent MN apoptosis using the MN1 cell line and primary cultures. Lentiviral expression of shRNA sequences targeting NgRs on these cells abolished protection by MAG-Fc. Analysis of RhoA activity using a FRET-based RhoA biosensor showed that MAG-Fc activates RhoA. Pharmacological inhibition of p75^NTR^/RhoA/ROCK pathway, or overexpression of a p75^NTR^ mutant unable to activate RhoA, completely blocked MAG-Fc protection against apoptosis. The role of RhoA/ROCK signaling was further confirmed in the nerve-crush model, where pretreatment with ROCK inhibitor Y-27632 blocked the pro-survival effect of MAG-Fc. These findings identify a new protective role of MAG as a modulator of apoptosis of MNs during postnatal development by a mechanism involving the p75^NTR^/RhoA/ROCK signaling pathway. Also, our results highlight the relevance of the nurture/protective effects of myelin on neurons.

A large number of motoneurons (MNs) undergo apoptosis during embryonic development.^1^ The most accepted theory to explain this process proposes that MNs are generated in large excess and compete for trophic support obtained predominantly via stable communication with their end-target organs.^2^ Developing MNs with limited trophic support degenerate by an apoptotic mechanism denoted programmed cell death involving activation of a pro-apoptotic signaling cascade via the low-affinity neurotrophin receptor p75^NTR^, which can be triggered by neurotrophins, including nerve growth factor (NGF).^3, 4^ At the membrane, p75^NTR^ exists in equilibrium between its homodimeric/monomeric forms, the former of which binds to NGF with high affinity.^5, 6^ Upon ligand binding, p75^NTR^ undergoes proteolytic cleavage, releasing its cytoplasmic death domain (DD), which in turn initiates a pro-apoptotic signaling cascade.^7^ In contrast, monomeric p75^NTR^ binds with low affinity to NGF but can act as a transducer molecule for other receptors. One example is the receptor complex formed by Lingo-1, Nogo-66 receptors (NgRs) and gangliosides, which upon activation increases its affinity for monomeric p75^NTR^ promoting the release of DD and activation of the small GTPase RhoA through displacement of Rho-GDP dissociation inhibitor α (Rho-GDIα).^5, 8, 9, 10^ RhoA signaling is the molecular switch for various extracellular signals and is known to mediate the regulation of diverse cellular processes, including apoptosis.^11, 12^ Survival of MNs during embryonic development relies on the activation of RhoA and its downstream effector Rho-associated kinase (ROCK).^13^ Also pharmacological activation of NgRs can antagonize p75^NTR^-dependent MN apoptosis in an *in vivo* model, highlighting the role of these receptors in early postnatal MN development.^14^

Myelin-associated glycoprotein (MAG) is a minor constituent of the nervous system selectively expressed at the periaxonal layer of myelinated axons.^15, 16^ MAG regulates axonal caliber, controls distribution of molecules at nodes of Ranvier, promotes axon stability and survival of neurons against excitotoxicity.^15, 17, 18, 19^ Its role as an inhibitor of axon regeneration had led to the discovery of its multiple neuronal receptors, including NgRs.^20^ In several types of neurons, the inhibitory action of MAG on axon regeneration relies in NgR/p75^NTR^-dependent RhoA/ROCK activation.^20, 21, 22, 23, 24, 25^ This led us to hypothesize that myelination could regulate postnatal development of MNs by modulating the pro-apoptotic activity of the p75^NTR^ receptor through binding of MAG to NgRs. Our results support a role for MAG as a modulator of apoptosis of MNs during postnatal development via interaction with NgRs and further activation of the RhoA/ROCK signaling pathway in a p75^NTR^-dependent manner.

## Results

### Mag-null mice display reduced numbers of MNs in the lumbar spinal cord (LSC)

**Figure 1:**
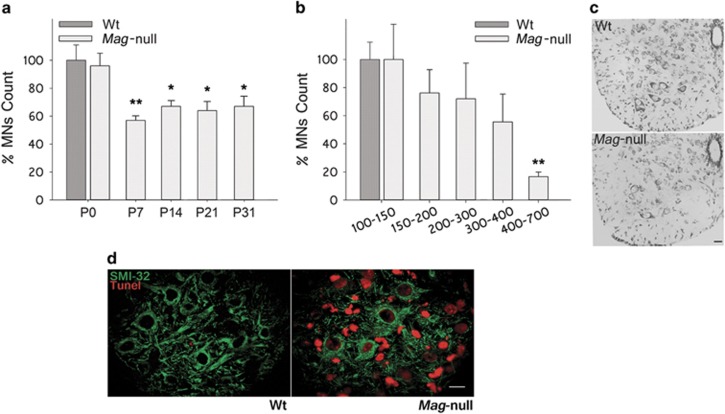
Reduced number of MNs in LSCs from Mag-null mice. (**a**) MNs from the LSC of *Mag*-null mice at postnatal days P0, P7, P14, P21 and P31 were counted and their numbers are expressed as a percentage of age-matched Wt control mice. MNs with an area ≥100 *μ*m^2^ were quantified using the Fiji software. (*P≤0.05, *n*=3 each group). (**b**) MNs at P31 were categorized by cell soma size as indicated. Cell counts for each size range in *Mag*-null mice are expressed as a percentage of cells of the same size range in Wt age-matched control mice. (**P≤0.01, *n*=3 each group). (**c**) Representative photomicrographs of LSC sections from Wt and *Mag*-null mice at P7, showing staining with cresil violet to identify neurons. Scale bar, 25 *μ*m. (**d**) Representative images of LSC sections from Wt and Mag-null mice at P3 depicting staining for apoptotic cells (TUNEL assay, red) and large caliber MNs (SMI-32 inmunostaining, green). Scale bar, 10 *μ*m

In order to study a possible role for MAG on the survival of MNs during postnatal development, we quantified the number of MNs in the LSCs from wild-type (Wt) and Mag-null mice. A time course study between postnatal days 0 and 31 (P0–P31) revealed that Wt and Mag-null mice had similar MN counts at P0 (Figure 1a). However, Mag-null mice displayed a ~43% reduction in MN counts at P7 with respect to Wt mice. MN counts in Mag-null mice at P14, P21 and P31 remained significantly lower than those in age-matched Wt control mice. Despite a similar distribution in the cell soma size of Mag-null and Wt mice at P0 (data not shown), Mag-null mice displayed a reduction of ~80% in the number of large MNs (cell soma >400 *μ*m^2^) with respect to Wt mice at P31 (Figures 1b and c). Similar observations were found when analyzing cell soma size in Mag-null mice at P7, P14 and P21 (data not shown). Further studies included analysis of apoptosis in P3-P4 LSC by TUNEL assay. Apoptotic cells could be only detected in LSC from Mag-null mice, some of them co-localizing by immunofluorescence with a specific marker for large size MNs (anti-non-phosphorylated neurofilament, mAb SMI-32) (Figure 1c). Overall, these data demonstrate that in the first postnatal week there is a significant increase in the apoptosis of large size MNs present in LSC of Mag-null mice with respect to Wt mice.

### Mag-null mice display increased susceptibility to apoptosis of MNs in an animal model of p75^NTR^-dependent apoptosis

**Figure 2:**
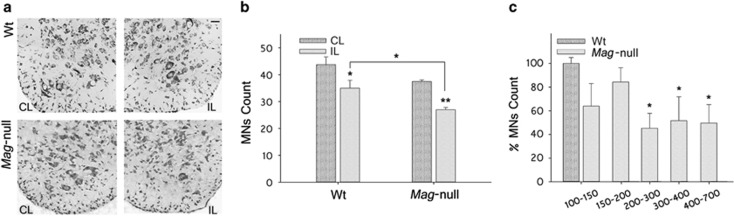
MNs from Mag-null mice are more susceptible to nerve-crush injury-induced apoptosis. P5 Wt and *Mag*-null mice received a contusion of the left sciatic nerve at mid-thigh level. Mice were killed at P10, and MNs≥100 *μ*m^2^ were quantified in cresyl violet-stained LSC sections using the Fiji software. (**a**) Representative photomicrographs of LSC sections from Wt and *Mag*-null mice stained with cresyl violet (Scale bar, 10 *μ*m). (**b**) Quantification of MNs 5 days after nerve lesion. Data are expressed as the MN count in ipsilateral (crushed) *versus* contralateral (control) LSC. (*P≤0.05, **P≤0.01, *n*=4 each group). (**c**) MNs count in the ipsilateral (crushed) LSC as a function of cell soma size. Data are expressed as the percent of MNs in *Mag*-null *versus* Wt mice. (*P≤0.05, *n*=4 each group)

We next studied the susceptibility of LSC MNs from Wt and Mag-null mice to apoptosis in an established *in vivo* model of p75^NTR^-dependent apoptosis induced by a sciatic nerve-crush injury at P5 followed by MN counts at P10. High-magnification images of Wt and Mag-null mice LSC showing the effect on MNs count of a nerve crush (ipsilateral) with respect to the contralateral size (intact nerve) are illustrated in Figure 2a. Nerve-crush injury on P5 Mag-null mice induced a reduction in MNs count respect to Wt mice (72±2.3% *versus* 80±3.4% MN count, respectively, Figure 2b). MN loss in Mag-null mice was selectively increased in larger-sized MNs with respect to Wt mice (Figure 2c). The data indicate that nerve-crush injury of P5 Mag-null mice induced increased death of MNs compared with Wt mice.

### MAG-Fc treatment rescues MNs in an *in vivo* model of p75^NTR^-dependent apoptosis

**Figure 3:**
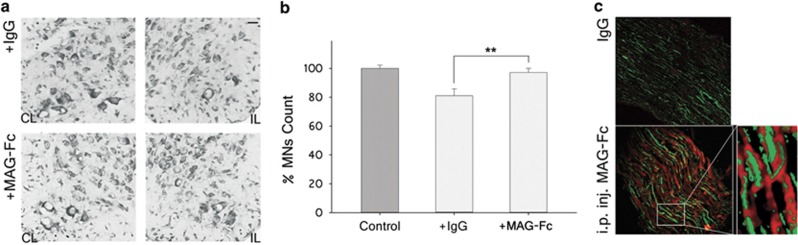
Mag-Fc protects MNs from p75^NTR^-dependent apoptosis in an *in vivo* model. Wt P3 pups were treated IP with 50 *μ*g of either MAG-Fc or control human IgG (*n*=4 mice each group). At P5, pups received a contusion lesion at mid-thigh level in their left sciatic nerve, and the survival of MNs was then analyzed at P10. MNs located in LSC with an area ≥100 *μ*m^2^ were quantified using the Fiji software. Comparison was made between LSC regions ipsilateral (crushed) *versus* contralateral (control) to the nerve-crush. (**a**) Representative photomicrographs of cresyl violet-stained sections of LSC from Wt mice receiving a contusion lesion and treated with MAG-Fc or control IgG (Scale bar, 10 *μ*m). CL: contralateral; IL: ipsilateral. (**b**) Quantification of MN count in the ipsilateral *versus* contralateral LSC of animals treated with MAG-Fc or control IgG (**P<0.01, *n*=4). (**c**) Representative photomicrographs shows the *Z*-projection image inmunostained for neurofilaments (clone 2H3, green) and human Fc γ chain contained in the MAG-Fc chimera (red) on sections from treated nerves with MAG-Fc or IgG control, acquired using confocal *Z*-reconstruction microscopy (Scale bar, 60 *μ*m)

To corroborate the regulatory role of MAG in apoptosis of MNs in the nerve-crush injury model, animals were treated with 50 *μ*g of MAG-Fc or control human IgG (IVIg) at P3 prior to nerve crush at P5. Quantification of MN survival at P10 indicated that MAG-Fc completely prevented lesion-induced apoptosis (Figure 3b). To identify a possible target for MAG-Fc's effect on MN survival, double immunofluorescence studies were performed on sciatic nerve sections. MAG-Fc-treated nerves receiving a contusion lesion showed specific immunostaining for human Fc *γ* chain contained in the MAG-Fc chimera restricted to axons, as evidenced by its colocalization with neurofilament staining (clone 2H3, Figure 3c). No signal was detected for human Fcs in nerves from IVIg-treated animals (Figure 3c). These data demonstrate that MAG-Fc treatment can rescue MNs *in vivo* from apoptosis possibly by binding to axons from MNs at the site of lesion.

### MAG-Fc protects MNs in *in vitro* models of p75^NTR^-dependent apoptosis

**Figure 4:**
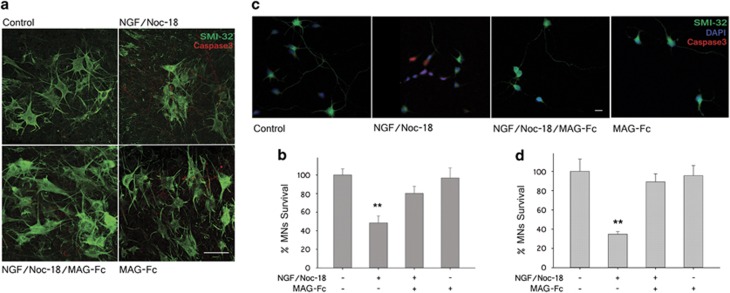
MAG-Fc prevents p75^NTR^-dependent apoptosis of MNs in *in vitro* models. SCOCs from P5 Wt mice and primary MNs cultures from E15 rats were grown for 7 and 5 days, respectively. p75^NTR^-dependent apoptosis was induced by treating cultures with 100 nM NGF plus 50 nM NOC-18 for 24 h (SCOCs) or 2 h (primary MNs). Some cultures were pretreated with 20 *μ*g/ml of MAG-Fc prior to the induction of apoptosis. (**a**) Representative confocal images illustrating survival of MNs in SCOCS from Wt mice; MNs were visualized with anti-SMI-32 mAbs (green) and apoptotic cells were identified by using anti-active caspase-3 mAbs (red) (Scale bar, 50 *μ*m). (**b**) MN survival in SCOCS from Wt mice (**P≤0.01; *n*=15). (**c**) Representative photomicrographs of primary MN cultures showing immunostaining for SMI-32 (green), active caspase-3 (red) and DAPI, 4,6-diamidino-2-phenylindole (blue) (Scale bar, 20 *μ*m). (**d**) MN survival (*P≤0.05; *n*=9)

A possible role of MAG on the survival of MNs was then tested in spinal cord organotypic cultures (SCOC) using an *in vitro* model of apoptosis that relies on the activation of p75^NTR^. In this model, MN death is induced by activation of the extrinsic and intrinsic pro-apoptotic pathways triggered by treatment for 24 h with low concentrations of NGF (100 nM) and NOC-18 (50 nM), a nitric oxide generator, respectively. In some cultures, treatment with MAG-Fc alone (20 *μ*g/ml) or combined with NGF/NOC-18 were carried out to test its efficacy against apoptosis. NGF/NOC-18 treatment induced a drastic reduction in MN survival in Wt SCOC (52±7%) while pretreatment with MAG-Fc largely abolished p75^NTR^-dependent apoptosis (Figure 4b). The effect of MAG-Fc against p75^NTR^-dependent apoptosis was tested on primary MN cultures (Figure 4c). Addition of NGF/NOC-18 to the cultures for 2 h resulted in a reduction of 65±3% in MN survival, which was completely prevented by pretreatment with MAG-Fc (20 *μ*g/ml) 4 h before induction of apoptosis. Similar results were obtained using a murine MN-derived cell line (MN1) where apoptosis was induced by treating differentiated MN1 cells as above (Figure 6g).

### MAG-Fc protects MNs from apoptosis via NgR receptors

**Figure 5:**
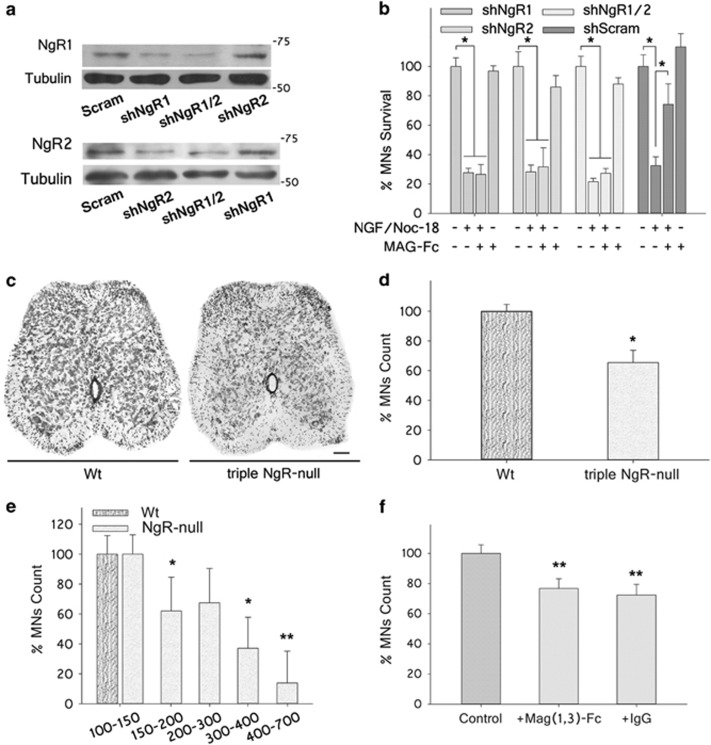
MAG-Fc protects MNs from p75^NTR^-dependent apoptosis via NgR receptors. (**a**) Western blotting showing NgR1 and NgR2 protein expression levels in MN1 cells transfected with rat NgR1 and NgR2 cDNAs for 4 days and further infected with lentiviral particles carrying shRNA sequences targeting NgR1 (shNgR1), NgR2 (shNgR2) or scrambled shRNA as control. Tubulin immunoreactivity was assayed to ensure equal amounts of loaded proteins. (**b**) Survival of NgR1- and NgR2-expressing MN1 cells 24 h after induction of apoptosis by addition of 100 nM of NGF plus 50 nM NOC-18 for 24 h. Cells were infected with shNgR1, shNgR2, shNgR1/2 or scrambled sequences as indicated. Some cultures were pretreated with 20 μg/ml of MAG-Fc (*P≤0.05; *n*=9). (**c**) Representative photomicrographs of LSC sections from Wt and triple NgR-null mice at P31 stained with cresyl violet (Scale bar, 50 *μ*m). (**d**) Quantification of MNs located in the LSC with an area≥100 *μ*m^2^ using the Fiji software (*P≤0.05; *n*=3 each group). (**e**) MNs at P31 were categorized by cell soma size as indicated. Cell counts for each size range in triple NgR-null mice are expressed as a percentage of cells of the same size range in Wt age-matched control mice (**P≤0.01, *P≤0.05, *n*=3 each group). (**f**) Wt P3 pups were treated IP with 50 *μ*g of either mutant form of MAG-Fc lacking extracellular domains 4&5 (MAG(1-3)-Fc) or control human IgG (*n*=3 mice each group). At P5, pups received a contusion lesion at mid-thigh level in their left sciatic nerve, and the survival of MNs was then analyzed at P10. MNs located in LSC with an area ≥100 *μ*m^2^ were quantified using the Fiji software. Comparison was made between LSC regions ipsilateral (crushed) *versus* contralateral (control) to the nerve-crush. Quantification (%) of MN survival in the ipsilateral *versus* contralateral LSC of animals treated with MAG(1-3)-Fc or control IgG (**P<0.01, *n*=3)

To determine the roles of NgRs in the protective effect of MAG-Fc on MNs, MN1 cells were infected with lentiviral particles carrying shRNA sequences targeting rat NgR1 (shNgR1) and/or NgR2 (shNgR2) inserted in a GFP-tagged plasmid to allow control of transfection. Cells were cultured for 4 days post infection to allow expression of shRNA sequences, and the role of MAG-Fc (20 *μ*g/ml) against apoptosis was then tested as described above. The specificity of shRNAs for silencing NgRs receptors was analyzed by western blotting. MN1 cells transfected with rat NgR1 and/or NgR2 sequences were infected with shNgR1 and/or shNgR2, which resulted in a robust and selective inhibition of NgRs (58% for shNgR1 and 43% for shNgR2; Figure 5a). MAG-Fc protection was completely abolished in MN1 cells infected with shNgR1 or shNgR2 with respect to control cells infected with a scramble sequence (Figure 5b). In addition, no significant additive effect on MAG-Fc protection was observed on cells infected with both particles (Figure 5b). These results highlight the critical contribution of both NgR1 and NgR2 to the signaling events triggered by MAG that inhibit apoptosis of MNs.

The role of NgRs on the survival of MNs was further tested using mice deleted of NgR1, NgR2 and NgR3 receptors (triple NgR-null). MNs present in the LSC from Wt and triple NgR-null mice were quantified at P31 as described. The data show a significant reduction in MNs surviving in triple NgR-null mice with respect to Wt mice (34.6±8.3%, Figure 5d). Analysis of cell soma size distribution showed a preferential reduction of large MNs (cell soma >300 *μ*m^2^) with respect to Wt mice (Figure 5e). The *in vivo* role of NgRs was further confirmed in the nerve-crush injury model by testing the efficacy of a soluble mutant MAG-Fc chimera (MAG(1-3)-Fc) lacking extracellular domains 4 and 5 required for activation of NgRs.^21, 26^ Treatment with MAG(1-3)-Fc failed to protect MNs when compared with IVIg (treated IP with 50 *μ*g at P3, *n*=3 each group). Double immunofluorescence studies on sciatic nerve sections from MAG(1-3)-Fc treated mice showed specific immunostaining for human Fc *γ* chain restricted to axons, evidencing binding to axonal receptor(s) (data not shown). Altogether these results confirm the *in vivo* relevance of these receptors in regulating the survival of MNs.

### MAG-Fc protects MN1 cells from apoptosis via activation of the RhoA/ROCK signaling pathway

**Figure 6:**
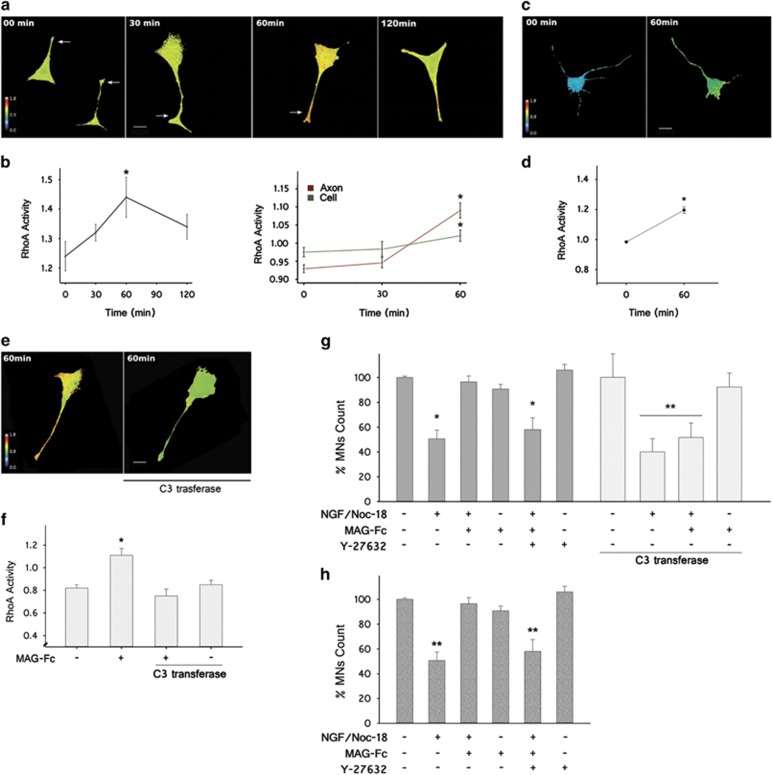
MAG-Fc protects MNs from p75^NTR^-dependent apoptosis via activation of RhoA/ROCK pathway. Time course of RhoA activation in MN1 cells and primary MN cultures after treatment with MAG-Fc. MNs were transfected with a FRET-based RhoA biosensor 18 h before treatment with 20 *μ*g/ml of MAG-Fc. RhoA activity is visualized as pseudo-color thermal map (warm colors, high FRET activity; cold colors, low FRET activity). (**a**) Images of MN1 cells captured at different times after MAG-Fc treatment illustrate RhoA activation on MN1 cells with a peak 60 min (Scale bar, 22 *μ*m). (**b**) Quantitative analysis of RhoA activity at different times after MAG-Fc treatment. The right graph shows a spatial analysis of RhoA activity in total cells and axons (green and red lines, respectively). Data represent the mean±S.E.M. of three independent experiments (*n*=15 cells per group, *P≤0.05). (**c** and **d**) Images of MAG-Fc-treated primary MN cultures depicting map of RhoA activity at 60 min and its quantification (*n*=12 cells per group) (*P≤0.05). (**e**) Images of MN1 cells co-transfected with RhoA biosensor and C3 transferase, an inhibitor of RhoA activity (as indicated) and further treated for 60 min with MAG-Fc. MN1 cells failed to show a MAG-Fc-dependent increase in RhoA activity (Scale bar, 20 *μ*m). (**f**) Quantitative analysis of RhoA activity of MN1 cells co-transfected with RhoA biosensor and C3 transferase and treated with MAG-Fc, data represent the mean±S.E.M. of three independent experiments (*n*=15 cells per group) (*P≤0.05). (**g**) Quantification of MN1 cell survival after transfection with C3 or treated with 10 nM Y-27632, an inhibitor of ROCK prior to the induction of apoptosis by treating cultures with 100 nM of NGF plus 50 nM NOC-18 for 24 h. Some cultures were treated with 20 *μ*g/ml of MAG-Fc. (*P≤0.05; *n*=9). (**h**) Quantification of primary MN cultures treated with 10 nM Y-27632 prior to the induction of apoptosis (see above) by treating cultures for 2 h. Some cultures were treated with 20 *μ*g/ml of MAG-Fc (**P≤0.01; *n*=3)

To determine the signaling pathways associated with the protective effect of MAG against apoptosis of MNs, we used a förster resonance energy transfer (FRET)-based RhoA biosensor to study its activity during MAG-Fc treatment. RhoA activity was calculated on MN1 cells 18 h after transfection with the biosensor and further treated with MAG-Fc (20 *μ*g/ml) at different times. MAG-Fc induced a robust increase in RhoA activity that started 30 min and peaked at 60 min after treatment. Spatial analysis showed that more activation occurred in the axon shaft and growth cone areas of MN1 cells compared with the cell soma (Figures 6a and b). Increased RhoA activity was also observed in primary MN cultures transfected with the RhoA biosensor (Figures 6c and d). The involvement of the RhoA signaling pathway was further confirmed by studying the effect of MAG-Fc on MN1 cells transfected with a plasmid containing a cytosolic form of C3transferase, a potent inhibitor of RhoA signaling (Figure 6e). MN1 cells transfected with C3 and RhoA biosensor failed to show a MAG-Fc-dependent increase in RhoA activity at 60 min (Figure 6f). C3 also rendered MN1 cells insensitive to the protective effect of MAG-Fc (Figure 6g). Similar results were obtained when MN1 cells were pretreated with 10 *μ*M Y-27632, a selective inhibitor of ROCK (Figure 6g). This result was replicated in primary MN cultures, where treatment with Y-27632 was able to block MAG-Fc protection against apoptosis. MNs expressing C3 or treated with Y-27632 without MAG-Fc treatment displayed a similar susceptibility to apoptosis induced by treatment with NGF/NOC-18 as untreated cells (Figures 6g and h).

### Treatment with Y-27632 blocks MAG-Fc protection on MNs in an *in vivo* model of p75^NTR^-dependent apoptosis

**Figure 7:**
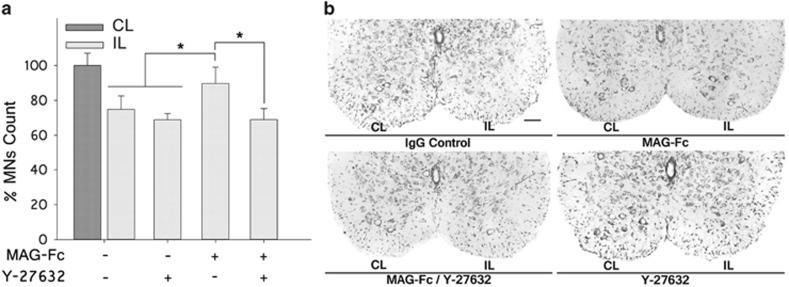
Treatment with Y-27632 blocks the protective effect of MAG-Fc in an animal model of MN apoptosis. P3 pups were treated IP with 50 μg of either MAG-Fc or control human IgG (*n*=6 each group). At P5, pups received a contusion at mid-thigh level of their left sciatic nerve. A group of animals treated with either control IgG or MAG-Fc were further treated with 1 *μ*l of Y-27632 (10 mg/ml, *n*=3) in the distal part of the crushed nerve close to the contusion area. Another group treated with control IgG (*n*=3, crush control) or MAG-Fc (*n*=3, control protection) did not receive further treatments. MNs located in the LSC with an area ≥100 *μ*m^2^ were quantified using the Fiji software. Comparison was made between LSC regions ipsilateral (crushed) *versus* contralateral (control) to the crush. (**a**) Bar graph shows MN counts on sections of LSC stained with cresyl violet (*P≤0.05 relative to Wt mice at the same age). Data are expressed as mean percentage±S.E.M. (**b**) Representative microphotographs of sections of ventral LSC showing the effect of Y-27632 on MAG-Fc protection against apoptosis of MNs. IP, ipsilateral; CL, contralateral

The involvement of the RhoA signaling pathway was further tested in the nerve-crush injury model. P3 pups were treated IP with 50 *μ*l of either MAG-Fc or IVIgG (1 mg/ml). At P5, pups received a contusion at mid-thigh level in their left sciatic nerve. A group of animals treated with either control IgG or MAG-Fc were further treated with 1 *μ*l of Y-27632 (10 mg/ml) injected into the distal part of the crushed nerve close to the contusion area. Another group treated with control IgG or MAG-Fc did not receive further treatments. MN counts in the LSC of P10 pups revealed that treatment with Y-27632 blocked the protective effects of MAG-Fc against lesion-induced apoptosis of MNs (Figure 7a). No significant differences were observed between mice receiving single treatments with control IgG or Y-27632, ruling out a possible toxic effect of the inhibitor on MNs. These data indicate that MAG-Fc protects MNs from p75^NTR^-dependent apoptosis *in vivo* by activating the RhoA/ROCK pathway.

### MAG-Fc protects MN1 cells from apoptosis via activation of RhoA/ROCK signaling pathway in a p75^NTR^-dependent manner

**Figure 8:**
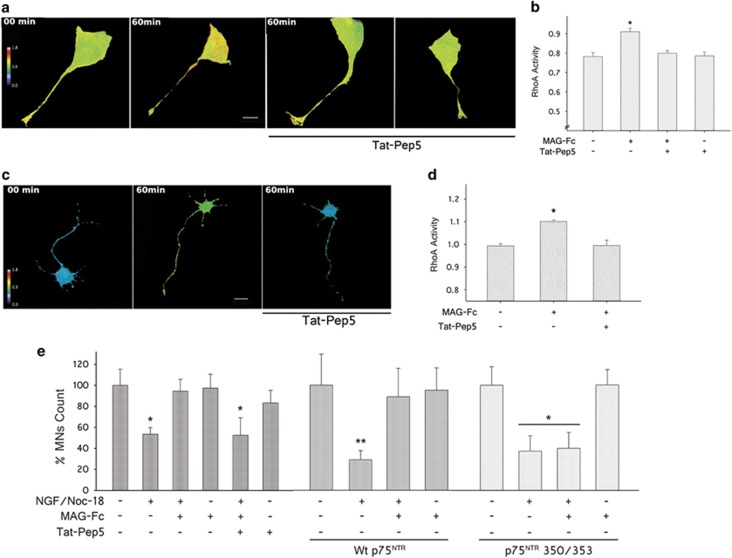
MAG-Fc protects MNs from apoptosis via p75^NTR^-dependent activation of RhoA/ROCK signaling pathway. RhoA activity in MN1 cells and primary MN cultures transfected with a FRET-based RhoA biosensor. After 18 h, some cultures were pretreated for 60 min with 200 nM TAT-Pep5, a specific inhibitor of p75^NTR^–RhoA interaction. Then MNs were treated with 20 *μ*g/ml of MAG-Fc for 1 h. (**a**) The images illustrate the pseudo-color thermal map of RhoA activation on MN1 cells (Scale bar, 22 *μ*m). (**b**) Quantitative analysis of RhoA activity on MN1 cells. Data represent the mean±S.E.M. of three independent experiments (*n*=15 cells per group) (*P≤0.05). (**c**) Images depict the pseudo-color thermal map of RhoA activation on primary MN cultures (Scale bar, 20 *μ*m). (**d**) Quantification of RhoA activity in MN cultures. Data represent the mean±S.E.M. of three independent experiments (*n*=12 cells per group; *P≤0.05). MN1 cells and primary MN cultures pretreated with TAT-Pep5 failed to show a MAG-Fc-dependent increase in RhoA activity. (**e**) Quantification of survival of MN1 cells pretreated with TAT-Pep5 or expressing Wt p75^NTR^ or its double mutant form 350/353 and further treated with 20 *μ*g/ml of MAG-Fc prior to the induction of apoptosis by treating cells with 100 nM NGF plus 50 nM NOC-18 for 24 h. (*P≤0.05; *n*=3)

As NGF and NgRs can signal through the p75^NTR^ receptor, experiments were performed to determine whether MAG-Fc modulates apoptosis of MNs by competing with NGF for the same transducer molecule. For this purpose, we tested the effect of TAT-Pep5, a cell-permeable inhibitor of NgR–p75^NTR^ interaction, on apoptosis of MNs. The efficacy of TAT-Pep5 to inhibit RhoA activity was tested on cells transfected with RhoA biosensor. MN1 cells pretreated with 200 nM TAT-Pep5 failed to show RhoA activation in response to MAG-Fc treatment (Figure 8b), and a similar result on RhoA activity was observed on primary MN cultures. On the other hand, TAT-Pep5 completely blocked MAG-Fc protection against apoptosis of MNs, ruling out the contribution of p75^NTR^-independent mechanisms (Figure 8e). Recently, two structural determinants involved in p75^NTR^–Rho-GDIα interaction from the cytosolic domain of p75^NTR^ have been described.^7^ Interestingly, these regions overlap with the region of DD recognized by TAT-Pep5. A double mutant form of DD located in these regions, displaying reduced affinity for Rho-GDIα (350/353), was unable to transduce the protective effect of MAG against apoptosis (Figure 8e), whereas treatment with MAG-Fc (20 *μ*g/ml) prevented apoptosis of MN1 cells transfected with Wt p75^NTR^. Overall, these data identify a common yet antagonic mechanistic link between the pro-apoptotic pathway induced by NGF and the anti-apoptotic pathway triggered by MAG that involves p75^NTR^.

## Discussion

The identities of molecules that contribute to the nurturing effects of myelin on neurons have emerged over the past several years. One such molecule is MAG, a minor component of the nervous system preferentially expressed on the periaxonal layer of myelinated axons. MAG regulates axonal caliber, controls the distribution of molecules at nodes of Ranvier and promotes axon stability under physiological conditions.^15, 17, 18, 19^ Its role as an inhibitor of axon regeneration led to identify its receptors and downstream signaling pathways.^20, 27, 28^ Recently, the axono-protective effects of MAG were confirmed in different models.^18, 19^ Interestingly, the protective role of MAG extends beyond ensheathed axons to neuron survival.^17^ In the present study, we identify MAG as a new regulatory component on the apoptosis of MNs during postnatal development. Previous work reported that pharmacological activation of NgRs resulted in protection of MNs against apoptosis.^14^ This led us to search for a regulatory signaling event through this receptor pathway. We identified that MAG, acting as a functional ligand of NgR1/NgR2 receptors, modulates postnatal apoptosis of MNs. Thus the protection exerted by MAG emerges as a critical factor to maintain survival of MNs during the first postnatal week, a period during which these neurons remain sensitive to deprivation of neurotrophins from their end-target organ.^29^ Interestingly, the anti-apoptotic signaling triggered by MAG via NgRs relies on p75^NTR^-dependent activation of the RhoA/ROCK signaling pathway.

p75^NTR^ is a molecule that pivots between pro-apoptotic and antiapoptotic signaling.^30^ During embryonic development, p75^NTR^ activation results in apoptosis of several neuronal populations, including spinal MNs.^30, 31^ Conversely, p75^NTR^-null mice displayed reduced apoptosis of spinal MNs.^32^ At the cell membrane, p75^NTR^ exists in equilibrium between its homodimeric/monomeric forms, both of which are active in signaling.^5, 6, 7^ Whereas neurotrophin-dependent activation of homodimeric p75^NTR^ is mostly associated with pro-apoptotic signaling, monomeric p75^NTR^ has been linked to a multimeric receptor complex, including NgRs, Lingo-1 and gangliosides, where it displays reduced affinity for NGF.^5^ Engagement of NgRs by MAG can halt axon regeneration via activation of the downstream signaling pathway RhoA/ROCK in a p75^NTR^-dependent manner.^9, 10, 20, 21, 24^ RhoA activation involves interaction of intracellular domains of p75^NTR^ with Rho-GDIα and further displacement of Rho-GDIα/RhoA complex.^9^ Although MAG activates RhoA signaling via NgR1 or NgR2, there are differences in the transducer molecule recruited by these receptors. NgR1-dependent activation of RhoA can be achieved via activation of p75^NTR^ or Taj/Troy receptors.^33, 34, 35, 36^ The transducer molecules for NgR2 remain elusive, although an NgR2/Troy interaction has been suggested.^37^ As our data support a functional role for both NgR1 and NgR2 in the regulatory role of MAG against MN apoptosis via interaction with p75^NTR^, several important questions arise. First, does binding of MAG to NgR1/p75^NTR^/Lingo-1 receptor complex shift the equilibrium of p75^NTR^ toward its monomeric form at the cell membrane? If that is the case, then MAG may modulate MN apoptosis via direct activation of this multimeric receptor while attenuating neurotrophin-induced pro-apoptotic activation via homodimeric p75^NTR^. In this sense, neurotrophin binding to p75^NTR^ is known to decrease RhoA activity by unknown mechanisms.^8^ Our work using inhibitors of RhoA/ROCK pathway combined with studies demonstrating the relevance of p75^NTR^–RhoA interaction provide evidence of a direct pro-survival effect of MAG on MNs via this signaling pathway. Also, the fact that the level of p75^NTR^-dependent apoptosis induced by NGF did not change significantly on control MN1 cells when silencing NgRs argues against the possibility of competing mechanisms. Although this question goes beyond the aims of the current study, future work may clarify how different p75^NTR^ ligands elicit distinct signaling responses. Second, does NgR2-dependent RhoA activation involve direct interaction with p75^NTR^? We found no additive effect on RhoA activation when silencing NgR1 and NgR2. Also inhibition of p75^NTR^–Rho-GDIα interaction abolished MAG-dependent RhoA activation, ruling out the possibility of p75^NTR^-independent mechanisms. Therefore, one possible explanation for our results is that MAG signals via interaction with a multimeric complex containing both NgR1 and NgR2 receptors. Future work will be required to identify the transducer molecules recruited by NgR2 after binding with MAG.

A third important question arises from the observation that the pro-survival effect of MAG on MNs requires activation of the RhoA/ROCK signaling pathway. A previous study using conditional expression of a dominant-negative form for RhoA and ROCK has found increased MNs apoptosis during embryonic development.^13^ Therefore, the survival role of RhoA/ROCK pathway triggered by MAG seems to recapitulate the developmental program of MNs. However, activation of RhoA/ROCK pathway is not always nurturing for neurons. One example is the postnatal development of the cerebral cortex, where RhoA/ROCK activation increases neuronal apoptosis.^38^ The opposite roles observed for RhoA/ROCK pathway ultimately reflect the complexity of signaling cascades regulating neuronal development. Of note, a neuroprotective pathway involving RhoA/ROCK activation in an NgR-dependent manner was recently reported.^39^ Whether similar molecular mechanisms downstream of RhoA are involved in MAG protection of postnatal MNs requires further investigation.

The use of RhoA and ROCK inhibitors has been successful to promote axon regeneration and/or functional recovery in many preclinical and some human clinical trials.^40, 41^ However, while in some cells ROCK can regulate caspase-3-dependent morphological changes during apoptosis, ROCK inhibition resulted in death in a variety of other cell types.^42^ The scenario becomes more complex when considering Rho effector proteins that works cooperatively with ROCK and proteins that modulate ROCK activity.^42, 43^ Thus ROCK may be pro-apoptotic or antiapoptotic depending on intrinsic properties of the cell and external environmental cues. Based on our work and the reports from literature, an important note of caution should be taken when considering the use of these inhibitors as therapeutic agents, in particular when considering the complexity of cell type-specific responses triggered by p75^NTR^ activation.

Although p75^NTR^ is widely expressed in the developing nervous system, in most cells p75^NTR^ expression is switched off at adult stages.^31, 44, 45, 46^ MNs are one of the few examples that retain low p75^NTR^ expression in the adult and, under certain injury/toxic insults, increase p75^NTR^ expression.^31, 47, 48^ Strong upregulation of p75^NTR^ expression in adult spinal MNs has been observed after peripheral nerve-crush injury in spinal cords of patients suffering from amyotrophic lateral sclerosis and animal models of this disease, although its biological significance in the context of a disease remains obscure.^4, 31, 48, 49^ Additionally, a nerve-crush injury in mice during the first postnatal week induces p75^NTR^-dependent MN death while a similar lesion in older mice results in nerve regeneration.^29^ The differential response to nerve-crush injury reflects the susceptibility of newborn MNs to restricted neurotrophin supply by end-target organs.^29^ In this sense, we described the loss of large MNs in *Mag*-null mice during the first postnatal week. Also, MAG was successful in preventing MN loss in a nerve-crush-induced model of apoptosis at this stage. The results reported here identify MAG as a critical regulatory component of postnatal MN development and, at the same time, highlight the relevance of the nurturing and protective effect that the process of myelination exerts on ensheathed neurons. Finally, this work could bring to light new pathogenic mechanisms underlying demyelinating/dismyelinating process associated with human diseases that, in addition to improving the understanding of these pathologies, could result in the development of therapeutic strategies to mitigate neurodegeneration.

## Materials and Methods

### Materials

Y-27632 (Rho kinase inhibitor) was from Calbiochem, Darmstadt, Germany. NEP1-40 (NgR-blocking peptide) and TAT-Pep5 (p75 NTR inhibitor) were from EMD Biosciences, La Jolla, CA, USA.^9, 50^ MAG-human Fc chimera (MAG-Fc) or a mutant form lacking extracellular domains 4/5 (MAG(1–3)-Fc) were purchased from R&D Systems, Minneapolis, MN, USA or were overexpressed in mammalian cells using a vector stably transfected into Flp-In-CHO cells (Invitrogen, Carlsbad, CA, USA) and then purified from culture supernatant by Protein-G chromatography and dialyzed against Dulbecco's phosphate-buffered saline (PBS).^51^ Their biological activity was confirmed by screening their binding to ganglioside GT1b by ELISA.^52^ Hank's balanced salt solution (HBSS), minimal essential medium (MEM), penicillin/streptomycin solution, Leibowitz's L15 medium, high-glucose Dulbecco's-modified Eagle's medium (DMEM), Lipofectamine 2000, OptiMEM transfection medium and fetal bovine serum (FBS) were purchased from Invitrogen, Grand Island, NY, USA. B27 supplement, heat-inactivated horse serum (HI-HS) and Neurobasal medium were purchased from Gibco. NOC-18 (nitric oxide generator), Optiprep solution, Mowiol, poly-D-lysine, all culture media supplements, salts and detergents were purchased from Sigma (Saint Louis, MO, USA).

The monoclonal antibody 12G10 (anti-alpha-tubulin) generated by investigators Frankel and Nelsen was obtained from the Developmental Studies Hybridoma Bank developed under the auspices of the NICHD and maintained by The University of Iowa, Department of Biology, Iowa City, IA, USA.

### Animals

*Mag*-null founder mice, kindly provided by Dr. Bruce Trapp, The Cleveland Clinic Foundation, Cleveland, OH, USA were constructed by disruption of exon 5 of the Mag gene as previously reported.^53^ Mutant mice were repeatedly back-crossed onto a C57BL/6 background to obtain 99.5% strain purity.^54^ Comparisons were made between age-matched *Mag*-null mice and C57BL/6 Wt mice. Triple NgR-null mice were created as described and were kindly supplied by Andrew Wood, Wyeth Research, Philadelphia, PA, USA.^55^ Comparisons were made between age-matched *Mag*-null mice and C57BL/6 Wt mice. Johns Hopkins University Animal Care and Use Committee approved of the work reported (Protocol Number MO11M83). All procedures were consistent with US federal law and NIH regulations. All of the experimental protocols developed at INIMEC-CONICET-Universidad Nacional de Córdoba were approved by the appropriate institutional animal care and use committees following the National Institutes of Health guidelines for the care and use of laboratory animals.

### Organotypic spinal cord cultures

LSCs were removed from 5-day old mice and placed in dissection media consisting of HBSS, sodium bicarbonate (4.3 mM), Hepes (4-(2-hydroxyethyl)piperazine-1-ethanesulfonic acid, 10 mM), D-glucose (33.3 mM), magnesium sulfate (5.8 mM), 0.03% bovine serum albumin and penicillin/streptomycin at 4 °C and sectioned (250 *μ*m) using a tissue chopper. Sections were transferred onto cell culture inserts (Millipore, Darmstadt, Germany) and cultured for a week in media consisting of 50% MEM supplemented with 25% HBSS, 25% HI-HS, Hepes (25 mM), D-glucose (35 mM), glutamine (2 mM) and penicillin/streptomycin. Cultures were maintained at 37 °C in a 5% CO_2_ incubator. The monoclonal antibody 12G10 (anti-alpha-tubulin) generated by investigators Frankel and Nelsen was obtained from the Developmental Studies Hybridoma Bank developed under the auspices of the NICHD and maintained by The University of Iowa, Department of Biology, Iowa City, IA, USA.

### Culture of primary MNs

Pregnant Wistar rats were anesthetized with CO_2_, and primary spinal cord cultures were prepared from E15 pups.^56^ Briefly, whole LSCs were excised and chopped in ice-cold HBSS. Tissue was then transferred in L15 medium and centrifuged at 1000 r.p.m. for 2–3 min. The spinal cords were enzymatically treated by incubating in prewarmed HBSS containing 0.25% trypsin at 37 °C for 20 min. Then, tissue was mechanically disaggregated by trituration with a fire-polished glass Pasteur pipette. Dissociated cells were placed on top of a 9% Optiprep solution in L15 medium (one tube with 3 ml of the Optiprep solution for every two embryos) and centrifuged at 2000 r.p.m. for 15 min. The top 2 ml of each gradient were carefully collected from each tube and combined. Cells were then rinsed twice with L15 medium at 1000 r.p.m. for 5 min to remove Optiprep. MNs were resuspended in a small volume of growing medium (250 μl) consisting of Neurobasal supplemented with albumin (2,5 mg/ml), catalase (2.5 *μ*g/ml), superoxide dismutase (2.5 *μ*g/ml), apo-transferrin (0.01 mg/ml), D-galactose (15 *μ*g/ml), progesterone (6.3 ng/ml), putrescine (16 *μ*g/ml), selenium (4 ng/ml), *β*-estradiol (3 ng/ml), hydrocortisone (4 ng/ml), biotin (2 ng/ml), L-glutamin (2 *μ*M) and B27. MNs were plated at low density (25–50 cells/mm^2^) on glass coverslips coated with 0.1 mg/ml poly-D-lysine and laminin 0.1 μg/ml and cultured for 3 days at 37 °C under 5% CO_2_.

### Sciatic nerve-crush model

A standardized mouse sciatic nerve-crush model was used.^14^ Five-day-old pups were hypothermically anesthetized, and their left sciatic nerves were crushed at mid-thigh level for 30 s with fine forceps. Separation of proximal and distal endoneurial contents indicated complete crush. Then skin incisions were sutured, and animals were allowed to recover. Treatments consisted of intraperitoneal (IV) injections with 50 *μ*l of MAG-Fc, MAG(1–3)-Fc or control IgG (1 mg/ml) 3 days before crush. Other groups of animals were administered IV MAG-Fc (same dosage) and Y-27632 (1 *μ*l, 10 mg/ml), a ROCK inhibitor, at lesion site immediately after nerve-crush injury. Spinal cords were collected 5 days after crush and processed for histological analysis as described below.

### Spinal cord histology and neuron counts

Mice were anesthetized with isoflurane and perfused transcardially with 4% paraformaldehyde in 0.1 M phosphate buffer. The complete spinal cord was removed and the lumbar region (L1–L5) was isolated and embedded in OCT. Serial cross-sections of LSCs at 10-*μ*m thickness were obtained in a cryostat, for a total of 52 serial sections (520-*μ*m total length) and processed for cresyl violet staining. Images were acquired using a light microscope (Zeiss, Jena, Germany) equipped with a Leica LC200 video camera (Heerbrugg, Switzerland), × 20 magnification, 1.42 NA air objective. Every fifth section in the anterior gray matter (left or right) was examined for the presence of MNs, which were identified by the presence of a large single nucleolus located within the nucleus and a cell soma area >100 *μ*m^2^. Gamma MNs range from 100 to 250 *μ*m^2^, whereas the larger alpha MNs range from 250 to 1100 *μ*m^2^.^57^ Cell counts and morphometry were accomplished using the Fiji image analysis platform (v.1.45, NIH, Bethesda, MD, USA).

### Culture of MN1 cell lines

Hybrid cell lines derived of mouse embryonic MNs (a gift of Dr. Ahmet Hoke, Johns Hopkins University, Baltimore, MD, USA) were grown in DMEM with 10% FBS and 2 mM glutamine at 37 °C in a 5% CO_2_ humidified atmosphere as described.^58^ Cells were grown on 96-multiwell plates or on glass coverslips coated with 0.1 mg/ml poly-D-lysine. Differentiation was induced by reducing the FBS concentration to 5% for 3–4 days.

### Induction of p75^NTR^-dependent apoptosis

To induce p75^NTR^-dependent MN death, organotypic cultures and MN1 cells were treated with low concentrations of NGF (100 ng/ml) (Alomone, Jerusalem, Israel) and 50 nM NOC-18 for 24 h as described.^14^ In primary MN cultures, apoptosis was induced by treatment with 100 ng/ml of NGF and 50 nM of NOC-18 for 2 h. In protection assays, 20 *μ*g/ml of MAG-Fc was added to culture media 1 h before induction of apoptosis.

### Inmunofluorescence

Organotypic cultures were fixed for 1 h in 4% paraformaldehyde, washed three times with PBS and incubated for 2 h at room temperature with blocking solution (PBS, 5% normal HI-HS, 0.5% TritonX-100). After blocking, primary antibodies were added in antibody blocking solution (PBS, 1% normal horse serum, 0.3% Triton X-100) 24 h at 4 °C. MN1 cells were fixed for 30 min at 37 °C in 4% paraformaldehyde, 0.12 M sucrose and then incubated for 1 h at room temperature in blocking buffer (PBS, 5% normal horse serum, 0.1% Triton X-100). Primary antibodies antiactive Caspase-3 (Cell Signaling, Danvers, MA, USA; used at 1 : 500 dilution) or antineurofilament SMI-32 (Abcam, Cambridge, MA, USA; used at 1 : 1000 dilution) in blocking buffer were added for 24 h at 4 °C and then were detected using Cy2-goat anti-rabbit antibody used at 1 : 500 dilution (Jackson Immunoresearch, West Grove, PA, USA) or with biotinylated anti-mouse (1 : 500 dilution) and Cy3-Streptavidin (1 : 1000 dilution) (Jackson Immunoresearch), respectively. Tissues were mounted with Krystalon (Merck KGaA, Darmstadt, Germany). Primary MN cultures were fixed for 30 min in 4% paraformaldehyde/0.12 M sucrose, washed three times with PBS and incubated for 30 min at room temperature with the appropriate blocking solution: 1.25% bovine serum albumin in PBS, 0.05% Triton X-100, and 2% normal goat serum. Primary antibodies were incubated in antibody blocking solution (10% normal goat serum/1.25% bovine serum albumin/0.05% Triton X-100) for 24 h at 4 °C. Active Caspase-3 and neurofilament immunoreactivty were tested as mentioned above. Cultures were mounted with Mowiol.

### *In situ* detection of DNA fragmentation by TUNEL assay

The TUNEL assay was performed in 30-*μ*m cryostat tissue sections using the *in situ* Apoptosis Detection Kit with TMR red (Roche, Basel, Switzerland, Cat. no. 12156792910). The activation of endonucleases during apoptosis generates the cleavage of nuclear DNA into fragments free 3′-OH end. This method uses a TdTenzyme (terminal deoxynucleotidyl transferase) for the incorporation of triphosphate deoxynucleotides (dUTP) to DNA fragments with 3′-OH end (TUNEL reaction). Tissue sections from Wt and *Ma*g-null mice were incubated for 2 min on ice with blocking solution (PBS/0.1% Triton X-100/0.1% sodium citrate). Then, the TUNEL reaction mixture was added to tissue sections for 1 h at 37 °C in a humidified atmosphere in the dark. After this period, tissues were rinsed three times with PBS. Negative control was performed by omitting TdT. Brain sections from Wt mice receiving an intrastriatal injection of glutamate were used as a positive control. Tissues were mounted with Mowiol.

### Immunoblotting

Cultures of MN1 cells were homogenized in RIPA buffer containing 1% Triton X-100, 1% sodium deoxycholate, 0.1% SDS, 0.15 M NaCl, 0.05 M Tris-HCl, pH 7.2, containing protease inhibitor cocktail (Sigma, St. Louis, MO, USA, P8340). Homogenates were boiled for 5 min and sonicated for 20 s. After centrifugation (13 000 r.p.m., 10 min, 4 °C), the protein content of the supernatant was determined using the BCA assay (Pierce, Rockford, IL, USA). Cell homogenates were diluted in Laemmli buffer and separated on a 12% SDS-polyacrylamide gel (40 μg/lane). Proteins were blotted to nitrocellulose and submitted to reversible staining with 0.1% Ponceau S in 5% acetic acid to ensure homogeneous transfer. Immunoblotting was performed with polyclonal IgG rabbit anti-NgR1 and anti-NgR2 antibodies diluted 1 : 2000 (kindly provided by Dr. Roman Giger, University of Michigan, USA) and detected using Immun-Star horseradish peroxidase-conjugated goat anti-rabbit diluted 1 : 3000 (Bio-Rad, Hercules, CA, USA).^26^ To ensure that equal amounts of protein were loaded, tubulin immunoreactivity was determined under the same conditions as described, using a monoclonal anti-alpha-tubulin mAb (clon 12G10) diluted 1 : 1000 and horseradish peroxidase-conjugated goat anti-mouse IgG diluted 1 : 5000 (Jackson Immunoresearch). Membranes were developed using enhanced chemiluminescence.

### Production of lentiviral vectors and infection of MN1 cells

shRNA sequences for NgR1 and NgR2 were obtained of Sigma-Aldrich (St. Louis, MO, USA, Mission shRNA). These sequences were ligated to the eGFP-expressing lentiviral vector pLKO.3G (Addgene, Cambridge, MA, USA) via sites EcoRI and PacI. We used as control shRNA ‘scrambled' sequences designed using the siRNA Wizard Program (InvivoGen, San Diego, CA, USA). Oligonucleotide shRNA sequences were as follows: NgR1: 5′-AATTCTCTACCTACAAGACAACAAT-3′, NgR2:5′-AATTGGTCAGCCTACAGTACCTCTA-3′ and scrambled: 5′-CCTAAGGTTAAGTCGCCCTCGCTCG-3′. Lentiviral vectors were produced by transient cotransfection of human embryonic kidney 293T cells in DMEM containing 10% FBS, using the recombinant plasmid-pLKO.3G carrying transgene sequences, sequences encoding helper (packaging) PsPax2 (Addgene) functions and sequences encoding Env glycoproteins PMD2.G (Addgene). At 48–60 h postransfection, lentiviral vector stocks were concentrated by ultracentrifugation and titrated by flow cytometric (FACS) methods via infection of HEK 293 T cells.

Differentiated MN1 cells were infected by addition of lentiviral particles carrying shRNA sequences for NgR1 and NgR2 at 1 × 10^4^ pfu for 4 days. MN1 cells infected with the virus were identified by eGFP expression. Expression levels of the receptors were monitored by western blotting with receptor-specific antibodies.

### Expression plasmids and transfections

Full-length rat p75^NTR^ and its mutant form K350A/N353A (abbreviated 350/353) were expressed from a pcDNA3 vector backbone (Invitrogen) and were generously provided by Dr. Carlos F Ibañez (Karolinska Institute, Sweden). Transient transfections were performed in MN1 cells grown in 96-well culture dishes. Cells were transfected with 1.2 *μ*g/*μ*l of plasmid using Lipofectamine 2000 (0.4 *μ*l/well) in OptiMEM. The mixture was added to cells 72 h after plating. Cultures were fixed with 4% paraformaldehyde in 4% sucrose-containing PBS 18 h after transfection before being processed for immunofluorescence.

### Förster resonance energy transfer

Transient transfection with Wt Rho-A biosensor plasmid (Addgene cat. no.12150) was performed by incubating MN1 cells with 1.2 *μ*g of plasmid in Lipofectamine 2000 in a final volume of 500 *μ*l.^59^ Transient transfections on primary MN cultures of Rho-A biosensor plasmid was performed into 3 DIV MNs by mixing 0.8 μg/μl of DNA and 1.25 μl/glass (12 mm) of Lipofectamine 2000 in Neurobasal for 1 h. Cultures were fixed with 4% paraformaldehyde in 4% sucrose-containing PBS 18 h after transfection, mounted and processed for FRET map calculation as described. Cells were visualized through a Spinning Disk Olympus DSU microscope, using a 60 × magnification, 1.42 NA immersion objective. FRET map images reflecting RhoA activity in MN1 cells or primary MN cultures were calculated as described.^60^ Briefly, CFP and YFP images were acquired while exciting the donor. For emission ratio imaging, the following filter sets were used: 457 and 514 nm Argon multiline laser of 40 mw Model 35-IMA 040-220 of CVI MellesGriot (Carlsbad, CA, USA). Fiji software (open-source platform) was used to perform image analysis. All images were first shading-corrected and background-subtracted. The FRET image, because it had the largest signal-to-noise ratio and therefore provided the best distinction between the cell and the background, was thresholded to generate a binary mask with a value of 0 outside the cell and a value of 1 inside the cell. After multiplication by this mask, the FRET image was divided by the CFP image to yield a ratio image reflecting RhoA activation throughout the cell.

### Statistical analyses

Data analysis was performed using standard statistical packages (InfoStat System, Córdoba, Argentina). All values are shown as the mean±S.E.M. of at least three independent experiments. Student's *t*-test or one-way analysis of variance followed by Fisher's *post hoc* test was used to assess differences between MN axon calibers.

## Abbreviations

MAG: myelin-associated glycoprotein
IP: intraperitoneally
NgR: nogo receptor
MN: motoneuron
p75^NTR^: low affinity receptor for neurotrophins p75
Rho-GDIα: Rho-GDP dissociation inhibitor α
ROCK: Rho-associated kinase
LSC: lumbar spinal cord
FRET: förster resonance energy transfer
MAG-Fc: human MAG-Fc chimera
DD: p75^NTR^ cytoplasmic death domain
